# Identifying relevant EEG channels for subject-independent emotion recognition using attention network layers

**DOI:** 10.3389/fpsyt.2025.1494369

**Published:** 2025-02-10

**Authors:** Camilo E. Valderrama, Anshul Sheoran

**Affiliations:** ^1^ Department of Applied Computer Science, University of Winnipeg, Winnipeg, MB, Canada; ^2^ Department of Community Health Sciences, Cumming School of Medicine, University of Calgary, Calgary, AB, Canada

**Keywords:** emotion recognition, electroencephalogram, affective computing, deep learning, attention mechanism, EEG signal processing

## Abstract

**Background:**

Electrical activity recorded with electroencephalography (EEG) enables the development of predictive models for emotion recognition. These models can be built using two approaches: subject-dependent and subject-independent. Although subject-independent models offer greater practical utility compared to subject-dependent models, they face challenges due to the significant variability of EEG signals between individuals.

**Objective:**

One potential solution to enhance subject-independent approaches is to identify EEG channels that are consistently relevant across different individuals for predicting emotion. With the growing use of deep learning in emotion recognition, incorporating attention mechanisms can help uncover these shared predictive patterns.

**Methods:**

This study explores this method by applying attention mechanism layers to identify EEG channels that are relevant for predicting emotions in three independent datasets (SEED, SEED-IV, and SEED-V).

**Results:**

The model achieved average accuracies of 79.3% (CI: 76.0-82.5%), 69.5% (95% CI: 64.2-74.8%) and 60.7% (95% CI: 52.3-69.2%) on these datasets, revealing that EEG channels located along the head circumference, including *Fp*_1_, *Fp*_2_, *F*_7_, *F*_8_, *T*_7_, *T*_8_, *P*_7_, *P*_8_, *O*_1_, and *O*_2_, are the most crucial for emotion prediction.

**Conclusion:**

These results emphasize the importance of capturing relevant electrical activity from these EEG channels, thereby facilitating the prediction of emotions evoked by audiovisual stimuli in subject-independent approaches.

## 1 Introduction

Detecting emotions via electroencephalography (EEG) offers an objective method for quantifying emotional states, as individuals cannot consciously control their EEG signals like they can their facial expressions, body posture, or speech (1). This objectivity in measuring emotions can be valuable in various fields as it provides the opportunity to respond to someone’s emotional state, rather than the subjective body language. For instance, measuring emotional states can aid in diagnosing and treating mental disorders in healthcare, evaluating student engagement in education, and assessing customer reactions to advertisements in market research (2, 3).

The emotion recognition process involves extracting features from EEG signals to train artificial intelligence (AI) models to associate these features with distinct emotions. There are two different approaches to build models that recognize emotions from EEGs: subject-dependent and subject-independent. The subject-dependent approach trains and tests the emotion recognition models using EEG signals from the same individuals. In contrast, the subject-independent approach uses different individuals for training and testing. Models trained using the subject-independent approach are more practical, as new users can use them without requiring retraining (4). However, subject-independent models often yield lower performance than subject-dependent models due to the high variability in EEG signals among individuals (5–8). This problem is known as the domain shift problem in the field of machine learning, which arises when the assumption that training and test sets share the same distribution is violated (9).

The domain shift problem in EEG signals arises from the significant variability in brain signals among individuals. Consequently, the patterns learned from the training set often fail to generalize effectively to new individuals, resulting in reduced predictive performance. Previous studies have addressed this issue using the adversarial neural network approach, specifically the Domain-Adversarial Neural Network (DANN) (10). DANN aims to extract features that not only facilitate accurate task classification but are also invariant between the training and test sets (i.e., the source and target domains). Building on this idea, Özdenizci et al. (11) demonstrated that an adversarial learning framework enhances EEG-based emotion recognition in cross-subject and cross-session classification tasks. Similarly, Barmpas et al. (12) showed that incorporating DANN with convolutional neural networks effectively addresses inter-subject variability in EEG signals, resulting in more robust predictive models.

In addition to DANN, a potential way to enhance subject-independent approaches is to identify EEG channels that are consistently relevant across different individuals for predicting emotion (4). However, as the current practice for emotion recognition relies on deep learning models, identifying relevant EEG channels is obscured by the low interpretability of deep learning models (13). This challenge can be addressed by incorporating layers within deep learning models that reveal the features driving the predictions. One such layer is the attention network layer (or “attention mechanism”), which has been effective in natural language processing (NLP) applications for identifying key words in text classification (14). Using a similar approach for subject-independent emotion recognition could help determine which features receive more attention from the deep learning model in predicting emotions across various individuals.

Previous studies have shown that attention layers in emotion recognition can enhance performance by capturing essential information from EEG signals. For instance, Arjun et al. (15) demonstrated that attention layers can improve emotion recognition performance by capturing essential information from EEG signals. Li et al. (16) used an attention layer to identify the most important EEG channels for feature extraction. Similarly, Feng et al. (17) applied attention network layers to assign weights to spatial-temporal features from EEG channels, extracting relevant patterns for emotion prediction. Although these studies have shown the benefits of attention mechanism layers, their focus has been mainly on enhancing prediction performance on subject-dependent approaches, thus relegating the interpretability aspect that the attention mechanism layer can offer.

Other studies have attempted to identify relevant EEG channels by analyzing energy distribution based on differential entropy (DE) features across the cortex (18–22). According to these analyses, happy stimuli produce more activation in the temporal lobe, fearful emotions trigger lower activation in the occipital area, and neutral stimuli activate the parietal and frontal lobes (18). Additionally, happy stimuli tend to generate higher activation than other emotions, particularly in the temporal lobes (19, 21). Regarding relevant brain areas, the lateral temporal lobe and the prefrontal lobe are more active than other areas for emotion regulation (20). However, these studies conducted their analyses prior to training deep learning models, thus overlooking the patterns that emerge during the training process. Since these learned patterns are crucial for emotion prediction, analyzing them post-training could provide valuable insights into identifying the most relevant EEG features.

Before the advent of deep learning models, feature selection techniques were employed to identify relevant EEG channels. Apicella et al. (23) reviewed 115 studies and found that channels $F_{p_{1}}$ , $F_{p_{2}}$ , *F*_3_, and *F*_4_ are most relevant for detecting the valence of an emotion, while *P*_3_ and *P*_4_ are most informative for the arousal dimension. However, many of these studies used subject-dependent approaches, which limits the generalizability and reproducibility of their findings.

All these previous studies have contributed to identifying relevant EEG channels for emotion recognition. They have identified these EEG channels either by analyzing the feature distribution or by applying feature selection techniques to improve prediction performance. However, these studies also exhibit some limitations. Some have focused on analyzing features prior to training deep learning, thus ignoring the patterns learned by the models. Others have focused more on prediction rather than interpretation. Moreover, most of these studies have identified relevant channels using subject-dependent approaches. Therefore, there is still a need for more effort toward identifying relevant EEG channels in subject-independent settings. In a previous work (24), we showed that attention layers have the potential to identify relevant areas for emotion prediction. In this current study, we extend upon that by using a deep learning architecture containing attention network layers to dynamically weight spatial and temporal features based on their relevance for emotion prediction across individuals on three different datasets. The ultimate goal is to leverage attention mechanisms to identify EEG channel locations that contribute the most to emotion prediction.

The main contributions of this paper to EEG-based emotion recognition are summarized as follows:

- The use of attention mechanism layers to classify emotions across three independent datasets: SEED (25), SEED-IV (26), and SEED-V (27).
- The analysis of the attention weights extracted by the attention layers to identify relevant EEG channels for emotion recognition.
- Highlighting the critical role of EEG channels along the head circumference in predicting emotions elicited by audiovisual stimuli.

## 2 Materials and methods

### 2.1 Datasets: SEED, SEED-IV and SEED-V

This study used EEG signals from three publicly available datasets, namely SEED (25), SEED-IV (26) and SEED-V (27). These datasets contain data collected from right-handed students aged 20 to 24 from the Shanghai Jiao Tong University, all of whom had normal hearing, vision, and a stable mental state.

In all the datasets, audiovisual stimuli were used to evoke different emotions. The targeted emotions in SEED were negative, neutral, and positive. For SEED-IV, the targeted emotions were happiness, neutrality, sadness, and fear, while SEED-V included the same four emotions plus disgust. While the subjects were watching the video clips, their EEG signals were recorded using 62 channels, positioned according to the 10/20 EEG system, with a sampling rate of 1000 Hz.

The SEED dataset (25) consists of data collected from 15 participants, of whom 8 were female. Each participant underwent three experimental sessions. During these sessions, EEG data was recorded as subjects watched 15 movie clips designed to elicit negative, neutral, and positive emotional responses. In total, 15 EEG signals were collected for each stimulus, resulting in 45 EEG signals for each participant.

The SEED-IV (26) dataset comprises EEG recordings of 15 subjects (eight female). Each subject participated in three sessions, in which they observed six video clips per emotion, resulting in 24 video clips per session. As a result, each subject watched 72 video clips after finishing the three sessions.

The SEED-V (27), on the other hand, encompasses data collected from 16 subjects. Each subject participated in three sessions, watching 15 movie clips in each session (three videos for each emotion). Thus, 45 were collected for each subject.

### 2.2 EEG processing

To enhance computational efficiency, recorded EEG signals were downsampled to 200 Hz using an anti-aliasing decimation filter, ensuring a Nyquist frequency of 100 Hz. Then, to reduce noise and artifacts caused by blinking or muscular movements, the EEG signals were filtered using a Butterworth filter within the range of 0.5-50 Hz. The selection of this range was made to ensure the inclusion of brain frequency bands: delta (*δ*: 0.5 − 4 Hz), tetha (*θ*: 4 − 8 Hz), alpha (*α*: 8 − 12 Hz), beta (*β*: 12 − 30 Hz), and gamma (*γ*: 30 − 50 Hz).

### 2.3 EEG segmentation

The EEG signals were segmented into non-overlapping 4-second windows. This segmentation provided a frequency resolution of 0.25 Hz $\left(\frac{1}{4,\text{s}}\right)$ , enabling the capture of two full cycles of the lowest frequency of interest in the delta band (0.5 Hz).

As the video clips in SEED, SEED-IV and SEED-V differed in duration, the number of 4-second segments obtained for each recording was different. **Tables 1**–**3** show the number of segments obtained for each recording in the datasets. In the SEED dataset, the number of 4-second segments per recording was 55.6 for the negative class, 54.8 for the neutral class, and 58 for the positive class. In the SEED-IV dataset, the average number of 4-second segments per recording was 38 for the neutral class, 38 for the sad class, 34 for the fear class, and 29 for the happy class. In the SEED-V dataset, the average number of 4-second segments per recording was 41 for the neutral class, 53 for the sad class, 41 for the fear class, 33 for the happy class, and 34 for the disgust class. The overall average number of 4-second segments per recording across all emotion classes was 56 in SEED, 34 in SEED-IV and 40 in SEED-V.

**Table 1:** Number of 4-second segments extracted for each recording in the SEED dataset.

Recording	Negative	Neutral	Positive
1	51	58	58
2	59	46	48
3	59	54	66
4	58	58	59
5	51	58	59
6	51	58	58
7	59	46	48
8	59	54	66
9	58	58	59
10	51	58	59
11	51	58	58
12	59	46	48
13	59	54	66
14	58	58	59
15	51	58	59
Mean	55.6	54.8	58

Last row shows the average number of segments per emotion across all the recordings.

**Table 2:** Number of 4-second segments extracted for each recording in the SEED-IV dataset.

Recording	Neutral	Sad	Fear	Happy
1	32	42	23	49
2	40	52	22	35
3	38	42	12	35
4	36	27	64	12
5	28	54	17	28
6	43	42	44	34
7	36	25	55	34
8	53	44	27	34
9	34	15	46	20
10	27	49	60	12
11	45	44	36	10
12	37	19	46	24
13	41	42	32	48
14	44	45	23	26
15	45	23	16	63
16	22	26	39	19
17	38	51	14	28
18	39	41	39	17
Mean	38	38	34	29

Last row shows the average number of segments per emotion across all the recordings.

**Table 3:** Number of 4-second segments extracted for each recording in the SEED-V dataset.

Recording	Neutral	Sad	Fear	Happy	Disgust
1	59	46	24	18	36
2	17	66	74	64	35
3	58	60	43	43	38
4	16	59	47	32	31
5	57	30	24	14	60
6	46	54	23	29	19
7	41	72	16	13	22
8	18	57	71	59	21
9	55	32	51	29	44
Mean	41	53	41	33	34

Last row shows the average number of segments per emotion across all the recordings.

### 2.4 Spectral features

For each 4-second window segment, the power spectrum density (PSD) was computed using the Hilbert-Huang Transform (HHT). The HHT was selected over other transformations, such as the Fourier and Discrete Wavelet, because, as we previously showed, HHT can better handle the non-linear and nonstationary characteristics of EEG for emotion recognition (28).

The PSD was computed for each EEG channel, covering the frequency range from 0 to 50 Hz. Consequently, each 4-second window produced a 2D structure with dimensions 62×50. The first dimension (rows) represented the 62 EEG channels, while the second dimension (columns) represented the frequency values, ranging from 0.5 Hz to 49.5 Hz with a 1 Hz step.

To facilitate the training of the deep learning model, the 2D structures corresponding to the same video clips were stacked, forming a three-dimensional structure. The first dimension of this structure represented the number of concatenated matrices (i.e., the number of 4-second segments per video clip), while the other two dimensions represented the EEG channels and frequency values. Since the number of 4-second segments varied across recordings (see **Tables 1**–**3**), zero-padding was applied to equalize the dimensions of all 3D structures. As a result, structures of dimensions 66 × 62 × 50 for SEED, 63 × 62 × 50 for SEED-IV and 74 × 62 × 50 for SEED-V were obtained for each video clip.

After calculating the spectrum tensors for each video, the tensors for the same subject were concatenated. This resulted in a four-dimensional tensor for each subject, with dimensions of (45, 66, 62, 50) for SEED-IV, (72, 63, 62, 50) for SEED-IV and (45, 74, 62, 50) for SEED-V. The first dimension represented the number of video clips for each dataset. Each of these tensors was assigned a class label corresponding to the emotion associated with the video clip. The datasets were balanced, with 15 tensors per emotion in SEED, 18 tensors in SEED-IV and nine tensors per emotion in SEED-V.

### 2.5 Emotion recognition model

**Figure 1:**
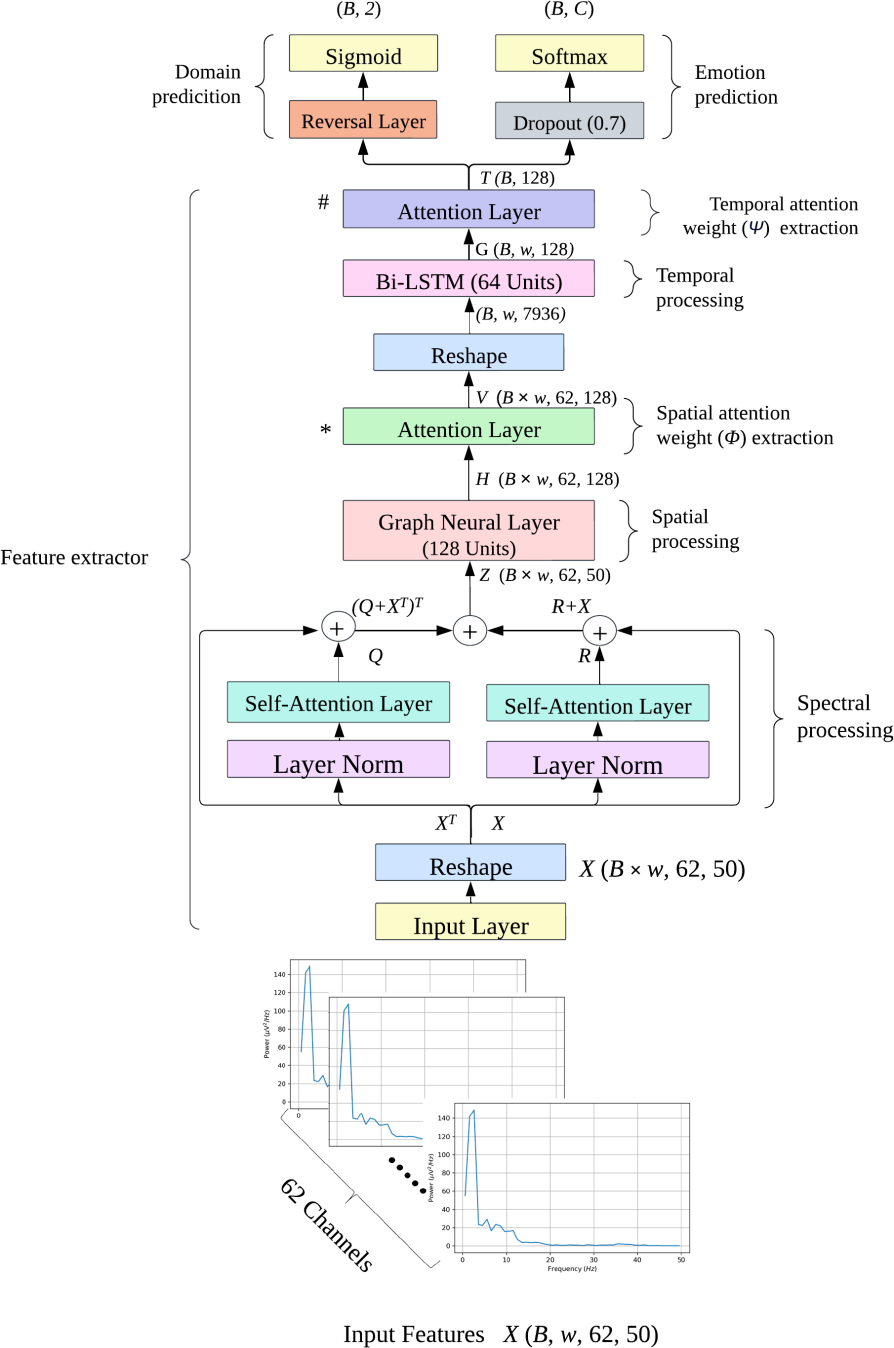
Emotion recognition model diagram. The input features are 4-dimensional tensors of shape (*B, w*, 62, 50), where *B* is the batch size, *w* is the number of windows (63 for SEED-IV and 74 for SEEDV), 62 is the number of EEG channels, and 50 is the frequency range. *C* denotes the number of emotion classes: 4 for SEED-IV and 5 for SEED-V. The attention layers, marked ∗ and # are where spatial and temporal attention weights are extracted, respectively. The model was trained using the DANN approach, where the extracted features were fed into both domain and emotion prediction models.

**Figure 1** shows the architecture of the deep learning models used to predict the behavior emotions from the spectral features. The input of this model had dimensions (*B, w*, 62, 50), where *B* is the batch size, *w* is the number of windows, 62 is the number of channels, and 50 is the number of frequencies. For SEED, *w* was 66, for SEED-IV, *w* was 63, and for SEED-V, *w* was 74. The batch size, *B*, was set to 64 for both datasets.

The deep learning model consisted of three modules. The first module aimed to further process the initial spectral features using self-attention layers to emphasize the frequencies and EEG channels that contribute the most to emotion prediction. The second module extracted spatial features using a graph neural layer (GNL), whereas the third module focused on extracting temporal features using a bidirectional long short-term memory (BI-LSTM) layer. To assess the relevance of the spatial and temporal features extracted by the GNL and BI-LSTM, these modules incorporated attention network layers after feature extraction.

To address the domain shift problem common in subject-independent emotion recognition, the deep learning model was trained using the domain adversarial neural network (DANN) approach. The DANN label predictor used the extracted spatial-temporal variables to predict the different emotions, while the DANN domain classifier used those features to distinguish between the training (source) and non-training (target) samples, attempting to find domain-invariant features for emotion prediction.

#### 2.5.1 Spectral processing

In the first module, we focused on emphasizing the spectral and spatial elements present in the power spectrum information of the input features. As the temporal dimension was not necessary at this point, we reshaped the input into a three-dimensional tensor of shape (*B* × *w*, 62, 50) by stacking the samples along the batch dimension, thereby preserving the EEG node and frequency dimensions of each tensor.

To further process the spectral information contained in the input tensors, we used two self-attention mechanism layers to compute the similarity between EEG channels based on their frequency values. The first self-attention layer aimed to enhance the frequency values by considering the correlation between EEG channels as follows:


(1) $$R=W_{channels}X,$$

where $W_{channels}$ was computed as $softmax\left(\frac{XX^{T}}{\sqrt{50}}\right)$ , indicating the correlation scores between each pair of EEG channels. *R* was an enhanced matrix in which the *j*-th frequency of the *i*-th EEG channel corresponded to the linear combination of the attention weights of the *i*-th EEG channel and the initial values for the *j*-th frequency across all EEG channels.

The second self-attention mechanism layer operated on the transpose of the spectral information to enhance the information of each EEG channel based on the correlation between frequencies. This was computed as follows:


(2) $$Q=W_{frequency}X^{T},$$

where $W_{frequency}$ was computed as $softmax\left(\frac{X^{T}X}{\sqrt{62}}\right)$ , indicating the correlation scores between each pair of frequencies. *Q* was an enhanced matrix in which the new values of the *j*-th frequency values of *i*-th EEG channel corresponded to the linear combination of the frequency weight scores and the initial frequency values of the *i*-th channel.

To promote stability during training, the inputs were first processed through a normalization layer before being fed into the self-attention layers. This normalization step helps keep the input feature distribution consistent. Furthermore, the output from the self-attention layers was combined with the original input, ensuring that the model preserved crucial information while improving feature representation and maintaining stability.

The outputs of the two self-attention mechanism layers were fused using an addition layer, as:


(3) $$Z=\left(\left(R+X\right)+{\left(Q+X^{T}\right)}^{T}\right).$$

This fusion allow us to autonomously learn and refine the feature representations extracted by the HTT transform.

#### 2.5.2 Spatial feature module

The second module used a GNL to correlate the spectral features of the EEG channels based on their location, thus generating spatial features. This was achieved as follows:


(4) $$H=ELU\left(SZW_{graph}\right),$$

where S was the adjacency matrix defined as $S={\tilde{D}}^{-\frac{1}{2}}\tilde{A}{\tilde{D}}^{-\frac{1}{2}}$ , where $\tilde{A}=A+I$ and $\tilde{D}$ is the degree matrix defined as $\tilde{D}i,i=\sum_{j}\tilde{A}ij$ . Here, I represents the identity matrix, and A is a 62-by-62 matrix, with each row and column corresponding to an EEG channel. The diagonal entries of A , $A_{i,i}$ , are set to 0, while the off-diagonal entries, $A_{i,j}$ , are set to the inverse of the Euclidean distance between the i -th and j -th EEG channels. $W_{graph}$ is a dense layer consisting of 128 units, using the ELU (Exponential Linear Unit) activation function.

The features extracted by the GNL were fed into an attention mechanism layer, aiming to identify the EEG channels whose features contributed the most to the prediction. Specifically, assuming that $h_{k,s}$ represents the feature vector of the *k*-th EEG channel at the *s*-th sample, the attention layer *G_b_* (·*,ω_b_*) projected $h_{k,s}$ into a hyperbolic space $u_{k,s}$ . Next, $u_{k,s}$ was fed into a softmax activation function to determine the normalized importance weight for each EEG channel, denoted as $\phi_{k,s}$ . These weights were then used to compute the context vector of EEG channel *v_k,s_* as:


(5) $$\begin{array}{l} u_{k,s}=\text{tanh}(W_{b}h_{k,s}+c_{b});\\ \phi_{k,s}=\frac{\text{exp}(u_{k,s}^{T}\cdot u_{b})}{\sum_{k}\text{exp}(u_{k,s}^{T}\cdot u_{b})};\\ v_{k,s}=\phi_{k,s}h_{k,s}. \end{array}$$

Each vector *v_k,s_* had dimension 128. All the *v_k,s_* vectors were arranged into a tensor *V* of dimensions (*B* × *w*, 62, 128).

#### 2.5.3 Temporal processing

To capture the variation of the extracted GNL features across time, the last stage used a BI-LSTM. To that aim, first we reshaped the dimensions of *V* from (*B* × *w*, 62, 128) to (*B, w*, 62 × 128), where *w* was 63 for SEED-IV and 74 for SEED-V. This reshaping allowed the features to be allocated in a temporally ascending order along the second dimension.

The number of units of the BI-LSTM was set to 64 units. As a result, the output of BI-LSTM had dimensions (*B, w*, 128), where 128 corresponds to the concatenation of 64 units from the forward LSTM and 64 units from the backward LSTM. This output encapsulates temporal information from both past and future contexts, making it highly informative for subsequent prediction tasks.

To identify the most relevant temporal features for emotion prediction, the outputs of the BI-LSTM were also fed into an attention layer (*G_a_* (·*,ω_a_*)), computing an attention weight $\psi_{w}$ and the final vector *t* as:


(6) $$\begin{array}{l} u_{w}=tanh\left(W_{a}g_{w}+c_{a}\right);\\ \psi_{w}=\frac{\text{exp}(u_{w}^{T}u_{a})}{\sum_{t}\text{exp}(u_{w}^{T}u_{a})};\\ t=\sum_{w}\phi_{w}g_{w}, \end{array}$$

where *g_w_* corresponds to the Bi-LSTM output at the *w_th_* segment, $\psi_{w}$ was the attention weight for the *w_th_* segment. The aggregated vectors *t* were arranged into a final tensor *T*, with dimensions (*B*,128).

#### 2.5.4 Domain adversarial neuronal network

Finally, the final feature vector, *T*, was fed into the label and domain classifiers of the DANN architecture. The label classifier consisted of a dropout layer with a rate of 0.7, followed by fully connected and softmax layers. The dropout layer was employed to mitigate overfitting, which is a common issue in subject-independent approaches. The softmax layer had four units for SEED-IV and five for SEED-V, outputting the probability of each sample belonging to a specific class.

The domain classifier included a reverse layer, followed by a dense layer with a single unit and a sigmoid activation function. This binary output indicated whether the sample was from the source domain (training set; class ‘0’) or to the target domain (test set; class ‘1’).

Following the DANN principles (10), the model was trained using the loss function defined as:


(7) $$\begin{array}{c} ℒ\left(\pi_{f},\pi_{y},\pi_{d}\right)=\frac{1}{n_{s}}\sum_{i=1}^{n_{s}}{ℒ}_{y}^{i}\left(\pi_{f},\pi_{y}\right)\\ -\lambda \left(\frac{1}{n_{s}}\sum_{i=1}^{n_{s}}{ℒ}_{d}^{i}\left(\pi_{f},\pi_{d}\right)+\frac{1}{N-n_{s}}\sum_{i=n_{s}+1}^{N}{ℒ}_{d}^{i}\left(\pi_{f},\pi_{d}\right)\right), \end{array}$$

where N was the total number of tensors, consisting of $n_{s}$ source tensors (training set) and $N-n_{s}$ target tensors (test set). The parameters $\pi_{f}$ , $\pi_{y}$ , and $\pi_{d}$ represent the parameters of the feature extractor, emotion predictor, and domain predictor modules, respectively. ${ℒ}_{y}^{i}$ and ${ℒ}_{d}^{i}$ represent the loss functions for label and domain predictions, respectively. The adaptation parameter λ was adjusted throughoutthe training epochs as:


(8) $$\lambda =\frac{2}{1+\exp\;(-10\times p)}-1,$$

where p was the training progress, which linearly varies from 0 to 1.

The parameters $\pi_{f}$ , $\pi_{y}$ , and $\pi_{d}$ were optimized using the following gradient updates:


(9) $$\begin{array}{l} \pi_{f}=\pi_{f}-\eta \left(\frac{\partial {ℒ}_{y}^{i}}{\partial \pi_{f}}-\lambda \frac{\partial {ℒ}_{d}^{i}}{\partial \pi_{f}}\right);\\ \pi_{y}=\pi_{y}-\eta \left(\frac{\partial {ℒ}_{y}^{i}}{\partial \pi_{y}}\right);\\ \pi_{d}=\pi_{d}-\eta \left(\frac{\partial {ℒ}_{d}^{i}}{\partial \pi_{d}}\right), \end{array}$$

where η was the learning rate. For the ${ℒ}_{y}^{i}$ , we used cross-entropy, whereas for the ${ℒ}_{d}^{i}$ , we used binary cross-entropy.

### 2.6 Experiment details

#### 2.6.1 Excecution environment

The models were implemented in TensorFlow2.0 and Python 3.10.1. We used a Colab account with 8 Intel(R) Xeon(R) CPU cores @ 2.30GHz, 12.7 GB of RAM, and 107.7 GB of hard drive space. The DANN architecture was trained using stochastic gradient descent (SGD) with a learning rate of 0.01 and a total of 100 epochs.

#### 2.6.2 Emotion prediction performance

To ensure a subject-independent approach, the model was evaluated using leave-one-out cross-validation (LOOCV). This means that during each iteration, samples from one subject were left out of the training process and used for testing instead.

For each iteration of the LOOCV, we calculated the performance for each emotion class using accuracy. Accuracy was determined by dividing the number of correctly predicted samples by the total number of samples in the class. The overall emotion accuracy was then computed as the average across all emotion classes.

#### 2.6.3 Ablation study

To evaluate the impact of each component on emotion prediction, we conducted an ablation study by training the model while excluding individual components of the deep learning architecture illustrated in **Figure 1**.

#### 2.6.4 Average spatial and temporal attention weights for emotion

After training the model for each subject, the spatial ( $\phi_{k,s}$ ) and temporal ( $\psi_{k,s}$ ) attention weights were extracted (see **Figure 1**). To visualize the spatial and temporal attention weights for each emotion class, we averaged these weights across subjects. This process aimed to identify EEG channels and 4-second segments with consistently higher values among subjects, thereby highlighting their relevance for emotion prediction. Specifically, for spatial attention, the attention weights of the *k*-th EEG channel at the *s*-th sample were averaged across all subjects ( $\phi_{k,s}$ ; Equation 5) for each emotion class. Similarly, for temporal attention, the attention weights at the *w*-th segment were averaged across all subjects ( $\psi_{w}$ ; Equation 6) for each emotion class.

#### 2.6.5 Identifying relevant EEG channels

To identify the relevant EEG channels to distinguish among emotions, we conducted statistical hypothesis tests to find significant differences in the attention weights extracted at the same EEG channel between emotions. To that end, all the spatial attention weight vectors ( $\phi_{k,s}$ ) and temporal ( $\psi_{w}$ ) corresponding to the same emotion were extracted for each subject. This resulted in a structure Φ of dimensions (*N_e_* × *w*, 62) containing all the spatial attention weights, and a structure (Ψ) of dimensions (*N_e_, w*) containing all the temporal attention weights, where (*N_e_*) is the total number of videos belonging to the emotion.

To aggregate the attention weights of the EEG channels across time, we computed the weighted average of the spatial weights based on the temporal weights. First, (Φ) was reshaped to dimensions (*N_e_, w*, 62) to separate the spatial weights for each 4-second segment. The aggregated weight for the *k*-th EEG channel at the *i*-th video for emotion *e* was calculated as:


(10) $$\omega_{i,k}^{e}=\sum_{w}^{\overset{¯}{W}}{\text{Φ}}_{i,w,k}\cdot {\text{Ψ}}_{i,w},$$

where ${\text{Φ}}_{i,w,k}$ and ( ${\text{Ψ}}_{i,w}$ ) represent the spatial and temporal weights, respectively, of the *i*-th video and the *k*-th EEG channel for the *w*-th segment and emotion *e*. $\bar{W}$ was the number of average 4-second windows for the dataset (56 for SEED, 34 for SEED-IV and 40 for SEED-IV). The reason for using the average number of segments is to ensure a consistent and fair comparison across the different emotions, as not all the videos have the same 4-second segments (**Tables 1**–**3**). All aggregated vectors were arranged into a matrix Ω*^e^* of dimensions (*N_e_*, 62).

The aggregated vectors of the videos and subjects were arranged into a structure Ω*^e^* of dimensions (*subjects* × *N_e_*, 62), where subjects were 15, 15, and 16 for SEED, SEED-IV, and SEED-V, respectively. To compare the activation patterns corresponding to each emotion, the overall weights of the EEG channels were analyzed using a two-sample Wilcoxon signed-rank test. This test assessed the null hypothesis that the distribution of the differences between the emotion pair *e*_1_ and *e*_2_ (e.g., sad vs. fear) was symmetric about zero, namely ${\text{Ω}}_{k}^{e_{1}}-{\text{Ω}}_{k}^{e_{2}}=0$ .

Given that three, six and ten possible emotion pairs were valid for SEED, SEED-IV and SEED-V, respectively, multiple hypothesis test were conducted. In detail, a total of 186, 372 and 620 comparisons were carried out for SEED, SEED-IV and SEED-V. To reduce false positive cases (Type I error), the p-values were adjusted using the Benjamini-Hochberg correction (29), setting the false-positive rate at 0.05.

## 3 Results

### 3.1 Emotion prediction

**Tables 4**–**6** show the performance achieved by each subject for different emotions in the SEED, SEED-IV, and SEED-V datasets, respectively. For all the datasets, the model surpassed the chance level accuracy, which is 33% for SEED, 25% for SEED-IV, and 20% for SEED-V. Specifically, for SEED, the average performance across all the subjects was 79.3%. In SEED-IV, the average performance across all subjects exceeded 60% for all emotions, achieving an overall accuracy of 69.5%. In contrast, for the SEED-V dataset, the average emotion accuracy was above 50% for all emotions, with an overall accuracy of 60.7%.

**Table 4:** LOOCV performance for emotion classification in the SEED datataset for each subject and emotion class.

Subject	Negative (%)	Neutral (%)	Positive (%)	Overall (%)
1	73.3	73.3	93.3	80.0
2	40.0	100.0	80.0	73.3
3	80.0	100.0	40.0	73.3
4	73.3	100.0	60.0	77.8
5	46.7	86.7	73.3	68.9
6	80.0	93.3	73.3	82.2
7	60.0	93.3	80.0	77.8
8	46.7	100.0	80.0	75.6
9	86.7	93.3	60.0	80.0
10	66.7	100.0	80.0	82.2
11	86.7	86.7	93.3	88.9
12	93.3	86.7	86.7	88.9
13	73.3	86.7	93.3	84.4
14	73.3	100.0	73.3	82.2
15	66.7	73.3	80.0	73.3
Mean (SD)	69.8 (15.7)	91.6 (9.2)	76.4 (14.4)	79.3 (5.8)
95% CI	61.1-78.5	86.4-96.7	68.4-84.4	76.0-82.5

The last column shows the overall performance across emotions. The final two rows display the mean and standard deviation (Mean ± SD) and the 95% Confidence Interval (CI) of performance for each emotion across the 15 subjects.

**Table 5:** LOOCV performance for emotion classification in the SEED-IV datataset for each subject and emotion class.

Subject	Neutral (%)	Sad (%)	Fear (%)	Happy (%)	Overall (%)
1	94.4	77.8	83.3	66.7	80.6
2	88.9	61.1	83.3	88.9	80.6
3	61.1	77.8	77.8	83.3	75.0
4	72.2	66.7	61.1	77.8	69.4
5	88.9	83.3	83.3	72.2	81.9
6	44.4	33.3	55.6	83.3	54.2
7	100.0	50.0	94.4	83.3	81.9
8	61.1	55.6	72.2	83.3	68.1
9	94.4	50.0	38.9	61.1	61.1
10	88.9	55.6	61.1	66.7	68.1
11	44.4	55.6	77.8	61.1	59.7
12	88.9	61.1	66.7	50.0	66.7
13	66.7	44.4	61.1	66.7	59.7
14	83.3	88.9	61.1	77.8	77.8
15	77.8	44.4	44.4	66.7	58.3
Mean (SD)	77.0 (17.9)	60.37 (15.8)	68.1 (15.5)	72.6 (11.0)	69.5 (9.6)
95% CI	67.1-87.0	51.6-69.1	50.6-76.7	66.5-78.7	64.2-74.8

The last column shows the overall performance across emotions. The final two rows display the mean and standard deviation (Mean ± SD) and the 95% Confidence Interval (CI) of performance for each emotion across the 15 subjects.

**Table 6:** LOOCV performance for emotion classification in the SEED-V datataset for each subject and emotion class.

Subject	Neutral (%)	Sad (%)	Fear (%)	Happy (%)	Disgust (%)	Overall (%)
1	88.9	66.7	55.6	77.8	77.8	73.3
2	88.9	33.3	22.2	77.8	55.6	55.6
3	88.9	44.4	77.8	88.9	55.6	71.1
4	88.9	33.3	88.9	77.8	55.6	68.9
5	77.8	55.6	66.7	55.6	33.3	57.8
6	77.8	33.3	11.1	44.4	66.7	46.7
7	44.4	66.7	22.2	66.7	66.7	53.3
8	100.0	66.7	77.8	100.0	77.8	84.4
9	55.6	22.2	66.7	100.0	77.8	64.4
10	33.3	33.3	11.1	11.1	33.3	24.4
11	11.1	55.6	66.7	100.0	55.6	57.8
12	44.4	77.8	33.3	44.4	44.4	48.9
13	100.0	44.4	66.7	66.7	44.4	64.4
14	44.4	55.6	33.3	44.4	44.4	44.4
15	100.0	55.6	77.8	55.6	100.0	77.8
16	88.9	88.9	77.8	88.9	44.4	77.8
Mean (SD)	70.8 (27.8)	52.1 (18.5)	53.5 (26.7)	68.8 (25.1)	58.3 (18.4)	60.7 (15.3)
95% CI	55.5-86.2	41.9-62.3	38.8-68.2	48.2-68.5	48.2-68.5	52.3-69.2

The last column shows the overall performance across emotions. The final two rows display the mean and standard deviation (Mean ± SD) and the 95% Confidence Interval (CI) of performance for each emotion across the 16 subjects.

In SEED, the neutral class achieved the highest performance, while the negative class achieved the lowest. For SEED-IV and SEED-V, the neutral and happy emotions achieved the highest accuracy, while the sad class had the lowest performance. Regarding variability among subjects, SEED-IV showed more consistent performance, with an overall standard deviation of 9.6%, compared to 15.3% for SEED-V. The highest variability in SEED-V was observed for subject 10, who achieved an overall accuracy of only 24.4%.

#### 3.1.1 Comparison with previous studies

**Table 7** presents a comparison of our model with previous emotion recognition models on the SEED, SEED-IV and SEED-V datasets using a subject-independent approach. Our proposed model achieved accuracy rates comparable to those of previous studies, attaining the second-best performance for SEED-IV and the sixth-best for SEED-V.

**Table 7:** Models comparison between previous emotion recognition methods and our approach (last row) of Models on SEED-IV and SEED-V Datasets.

Models	SEED	SEED-IV	SEED-V
SVM Suykens and Vandewalle (30)	56.7/16.2	37.9/12.5	23.71/8.2
DANN Kendall et al. (31)	–	47.6/10.0	–
BiDANN Li et al. (32)	83.2/9.6	65.6/10.4	–
BDGLS Wang et al. (33)	–	–	59.6/4.8
DGCNN Song et al. (34)	79.9/9.0	52.8/9.2	41.9/6.7
A-LSTM Song et al. (35)	72.1/10.8	55.0/9.3	40.3/08.7
P-GCNN Wang et al. (36)	–	–	64.8/9.8
IAG Song et al. (37)	86.3/6.9	–	59.7/9.4
RGNN Zhong et al. (38)	85.3/6.7	73.8/8.0	66.3/16.7
BiHDM Li et al. (39)	85.4/7.5	69.0/8.7	–
ECLGCNN Yin et al. (40)	–	–	61.6/10.4
GECNN Song et al. (41)	82.4/-	–	66.8/8.2
BiHDM w/o DA Li et al. (42)	81.5/9.7	67.4/8.2	–
PGCN Zhou et al. (43)	–	76.9/7.1	71.4/9.4
GMSS Li et al. (42)	86.52/6.22	73.48/7.41	–
Ours	79.3/5.8	69.5/9.6	60.7/15.3

Performance is reported as accuracy (mean average/standard deviation).

#### 3.1.2 Ablation study

**Table 8** shows the ablation study conducted by removing different components of the deep learning model shown in **Figure 1**. For all datasets, the component that resulted in the highest performance reduction was spatial processing, performed by the graph neural layer. Temporal processing and the temporal attention layer were also significant, leading to performance drops ranging from 0.5% to 9.5% and from 2.7% to 17.6%, respectively.

**Table 8:** Ablation study evaluating the removal of different components of the deep learning model shown in **Figure 1**.

Experiments	SEED		SEED-IV		SEED-V	
	Mean/SD (%)	Reduction(%)	Mean/SD (%)	Reduction(%)	Mean/SD (%)	Reduction(%)
Full model	79.3/5.8		69.5/9.6		60.7/15.3	
- spectral processing - EEG channel attention	75.7/07.2	4.5	69.2/7.9	0.5	59.4/10.6	2.2
- spectral processing - frequency bands attention	77.5/5.8	2.2	68.6/8.0	1.3	59.6/9.5	1.8
- spectral processing	76.3/7.4	3.7	69.2/12.3	0.5	56.8/9.1	6.4
- spatial processing - graph neural network	57.0/8.2	28.0	53.7/11.4	22.7	37.0/9.7	39.1
- attention layer spatial	78.1/5.0	1.5	69.4/10.7	0.1	58.1/7.6	4.2
- temporal processing	71.7/9.0	9.5	69.8/13.2	0.5	55.0/6.9	9.5
- attention layer temporal	65.3/5.2	17.6	67.6/13.5	2.7	50.5/8.3	16.7

### 3.2 Average spatial and temporal attention weights for emotion

#### 3.2.1 Average spatial attention weights for emotion

**Figure 2:**
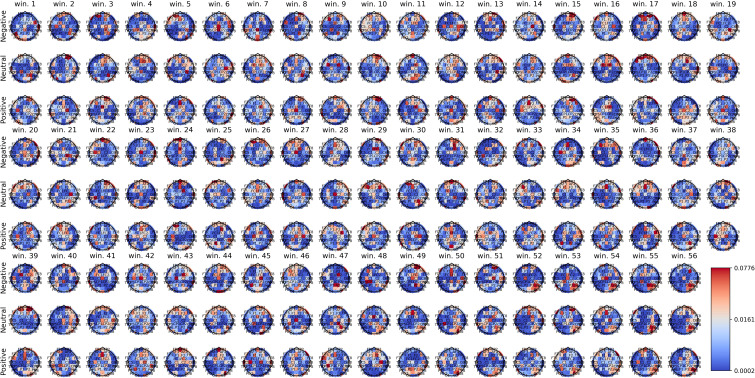
Average spatial attention weights ( ${\bar{\phi }}_{k,s}$ ) for each of the 62 EEG channels and each emotion across the 15 subjects over the initial 56 4-second segments of the SEED-IV dataset.

**Figure 3:**
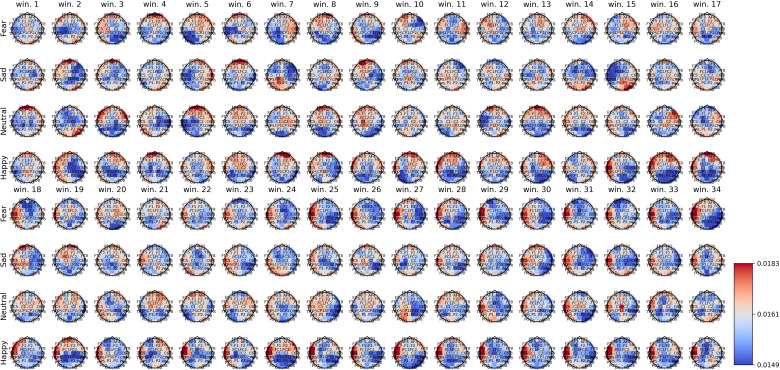
Average spatial attention weights ( ${\bar{\phi }}_{k,s}$ ) for each of the 62 EEG channels and each emotion across the 15 subjects over the initial 34 4-second segments of the SEED-IV dataset.

**Figure 4:**
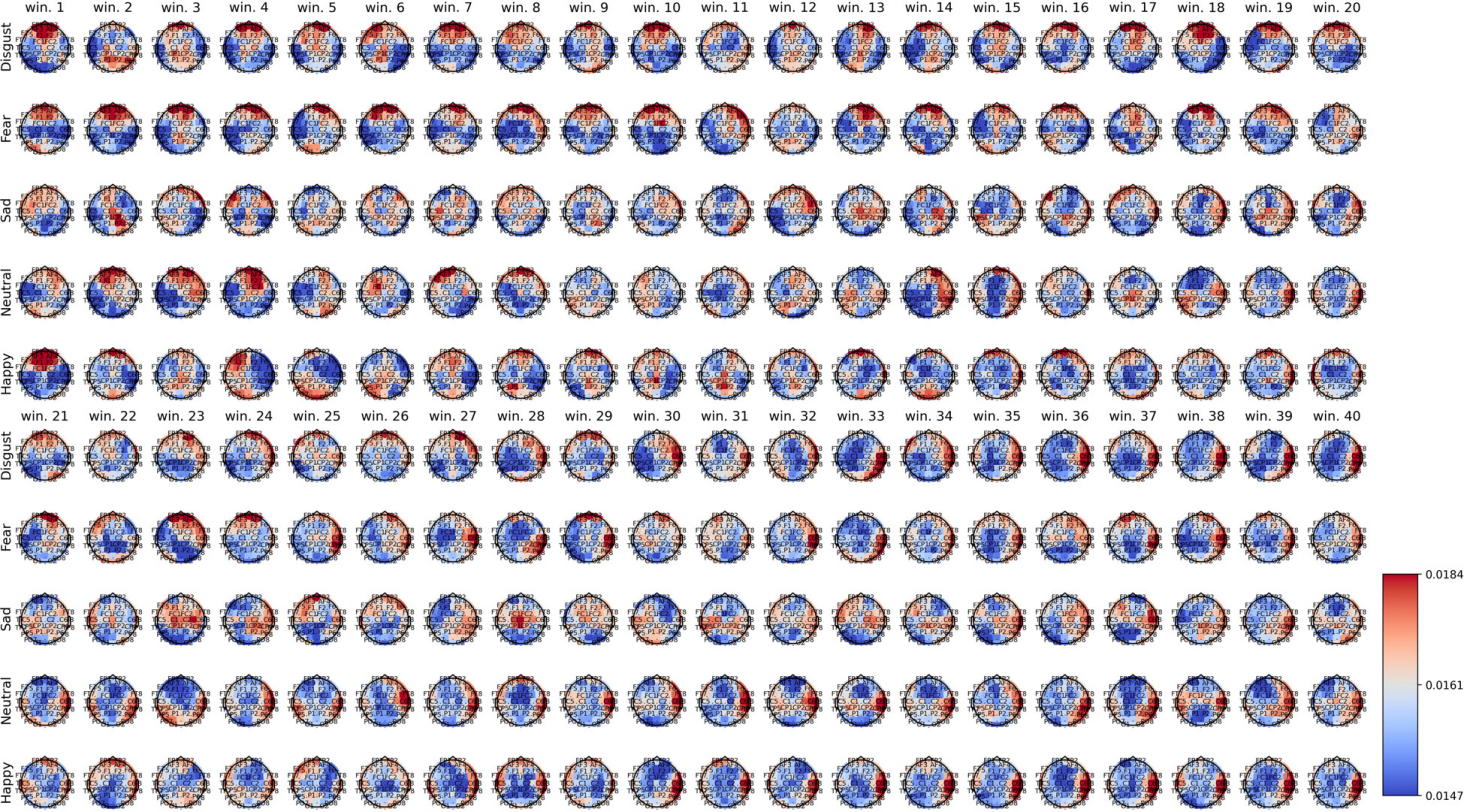
Average spatial attention weights ( ${\bar{\phi }}_{k,s}$ ) for each of the 62 EEG channels and each emotion across the 16 subjects over the initial 40 4-second segments of the SEED-V dataset.

**Figures 2**–**4** show the average spatial weights ( ${\bar{\phi }}_{k,s}$ ) extracted from the attention layer following the graph neural layer (GNL) for the average number of segments per dataset (56 for SEED, 34 for SEED-IV and 40 for SEED-V). In all the datasets, across all emotions, the spatial attention weights were higher around the frontal regions (*FP*_1_, *FP*_2_, *FP_Z_*, *F*_1_, *F*_2_, *F_Z_*). Spatial attention weights higher than the uniform weight (1/62) were also observed for EEG channels along the head circumference, particularly in the temporal and occipital regions. In comparison to the EEG channels located on the lateral sides of the temporal, frontal, and occipital areas, the EEG channels located in the central areas had weights lower than the uniform weight for most segments. The only classes that achieved higher weights for the central EEG channels were the sad and neutral classes.

**Supplementary Figures 1**–**3** show the average spatial attention weight distribution over all the 4-second segments (66 for SEED, 63 for SEED-IV and 74 for SEED-V). For SEED-I and SEED-V, the weights, after the average number of windows for the recordings (34 for SEED-IV and 40 for SEED-V), converged to a fixed pattern. For SEED-IV, this pattern consisted of higher weights along the left lateral frontal, temporal, and parietal regions. In contrast, for SEED-V, the pattern was the opposite, with high attention weights in the right lateral frontal, temporal, and parietal regions.

#### 3.2.2 Average temporal attention weights for emotion

**Figure 5:**
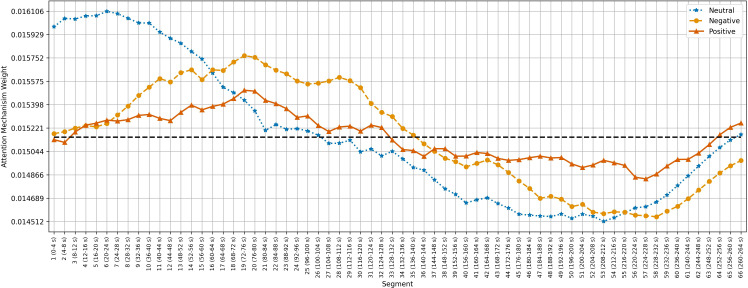
Average temporal attention weights ( ${\bar{\psi }}_{w}$ ) for each of 4-second segment and each emotion across the 15 subjects of the SEED dataset. The dotted line indicated the uniform weight (1*/*66 = 0.015).

**Figure 6:**
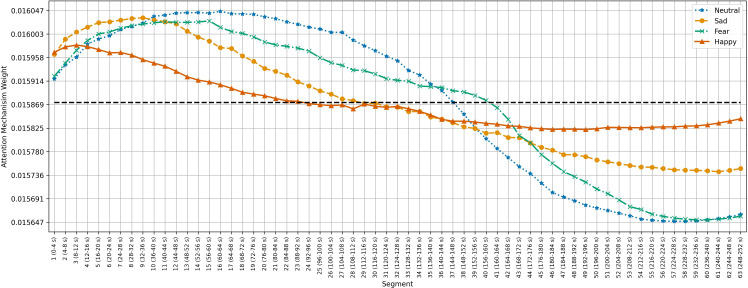
Average temporal attention weights ( ${\bar{\psi }}_{w}$ ) for each of 4-second segment and each emotion across the 15 subjects of the SEED-IV dataset. The dotted line indicated the uniform weight (1*/*63 = 0.015).

**Figure 7:**
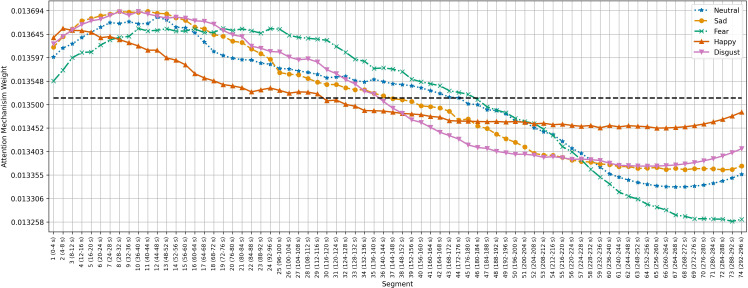
Average temporal attention weights ( ${\bar{\psi }}_{w}$ ) for each of 4-second segment and each emotion across the 16 subjects of the SEED-V dataset. The dotted line indicated the uniform weight (1*/*74 = 0.013).

**Figures 5**–**7** show the average temporal attention weights ( ${\bar{\psi }}_{w}$ ) for each 4-second segment in both datasets. The weights for segments beyond the average number of segments were lower than the uniform weight (i.e., 1*/*66 for SEED, 1*/*63 for SEED-IV and 1*/*74 for SEED-V), indicating that the predictive models relied little on the features extracted during the last time segments. For SEED-IV and SEED-V, the attention weights exhibited a concave parabolic trend: initially increasing steadily, reaching a maximum between the tenth and fifteenth segments, and then decreasing.

### 3.3 Identifying relevant EEG channels

**Figure 8:**
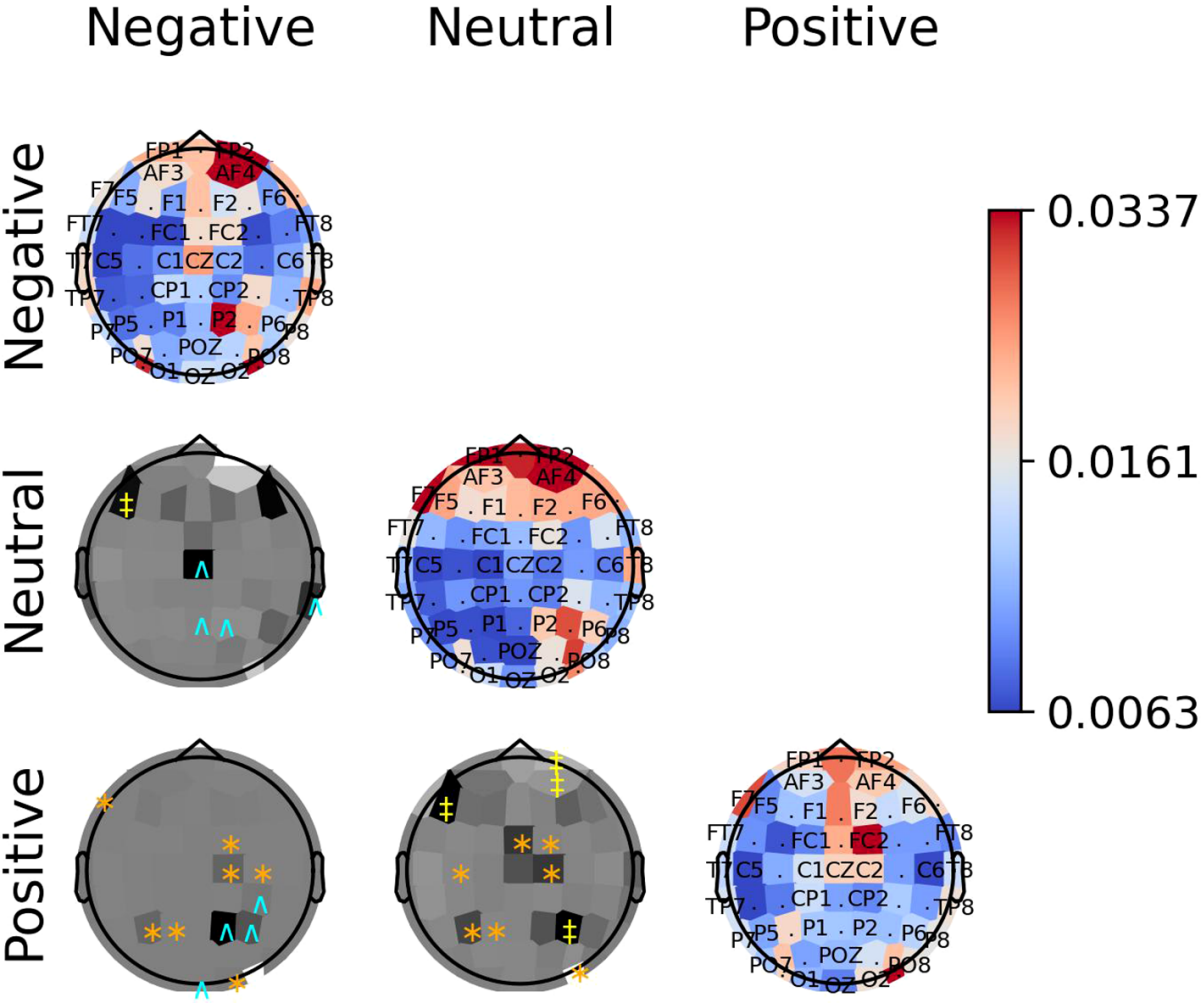
The diagonal panels shows aggregated attention weights obtained by the EEG channels for SEED dataset. The off-diagonal panels are the difference between aggregated attention weights obtained by the EEG channels. A darker color indicates a greater difference between the aggregated weights obtained for the EEG channel for the vertical and horizontal emotion pairs. Each symbol indicates that the weight difference between the emotion pair was significant (2-sided Wilcoxon rank-sum hypothesis tests adjusted via Benjamini-Hochberg correction with a false-positive rate set at 0.05) in favor of the class ‘negative’ (cyan ^∧^), ‘neutral’ (yellow ‡), or ‘positive’ (orange ∗).

**Figure 9:**
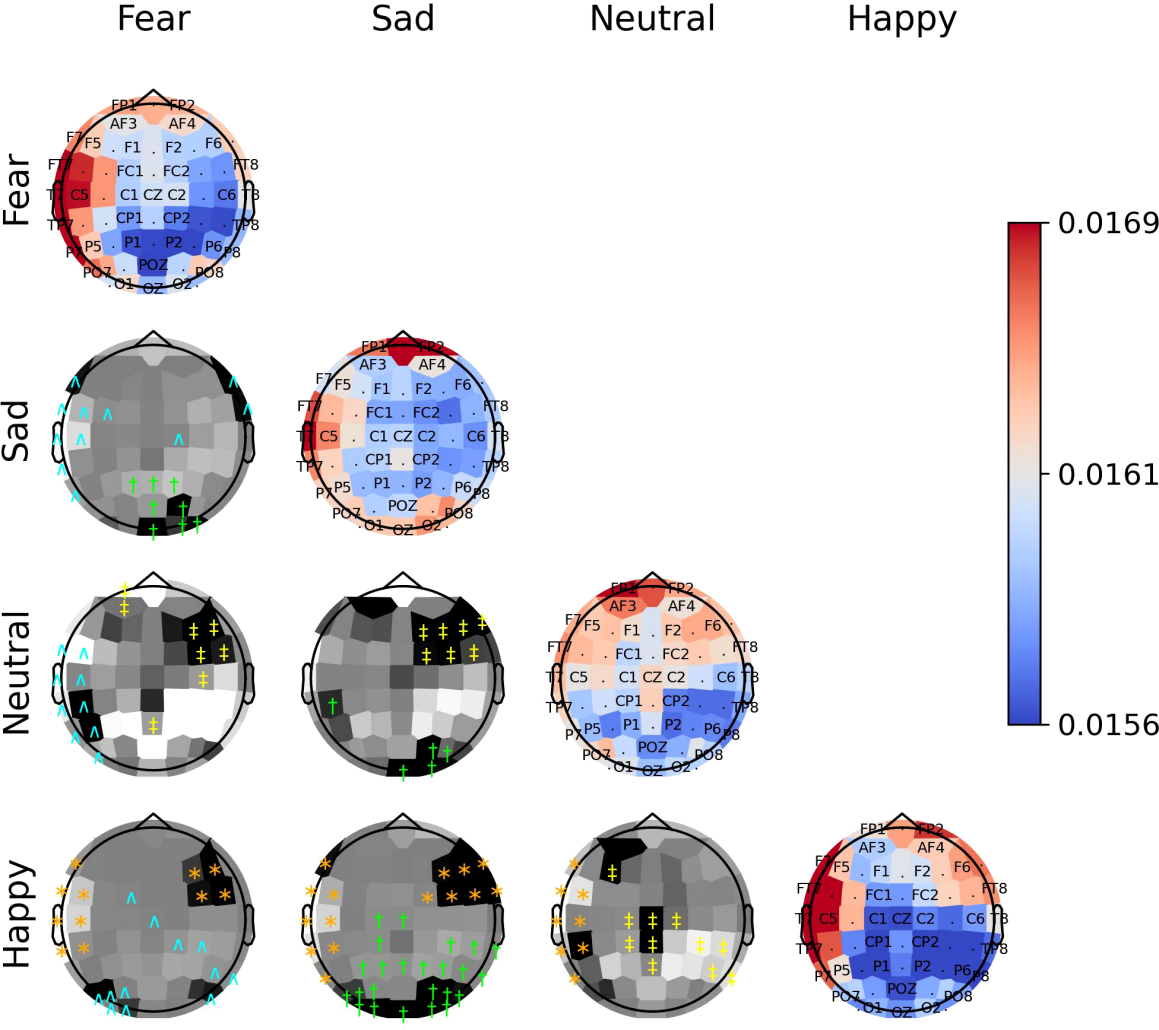
The diagonal panels shows aggregated attention weights obtained by the EEG channels for SEED-IV. The off-diagonal panels are the difference between aggregated attention weights obtained by the EEG channels. A darker color indicates a greater difference between the aggregated weights obtained for the EEG channel for the vertical and horizontal emotion pairs. Each symbol indicates that the weight difference between the emotion pair was significant (2-sided Wilcoxon rank-sum hypothesis tests adjusted via Benjamini-Hochberg correction with a false-positive rate set at 0.05) in favor of the class ‘fear’ (cyan ^∧^), ‘sad’ (green †), ‘neutral’ (yellow ‡), or ‘happy’ (orange ∗).

**Figure 10:**
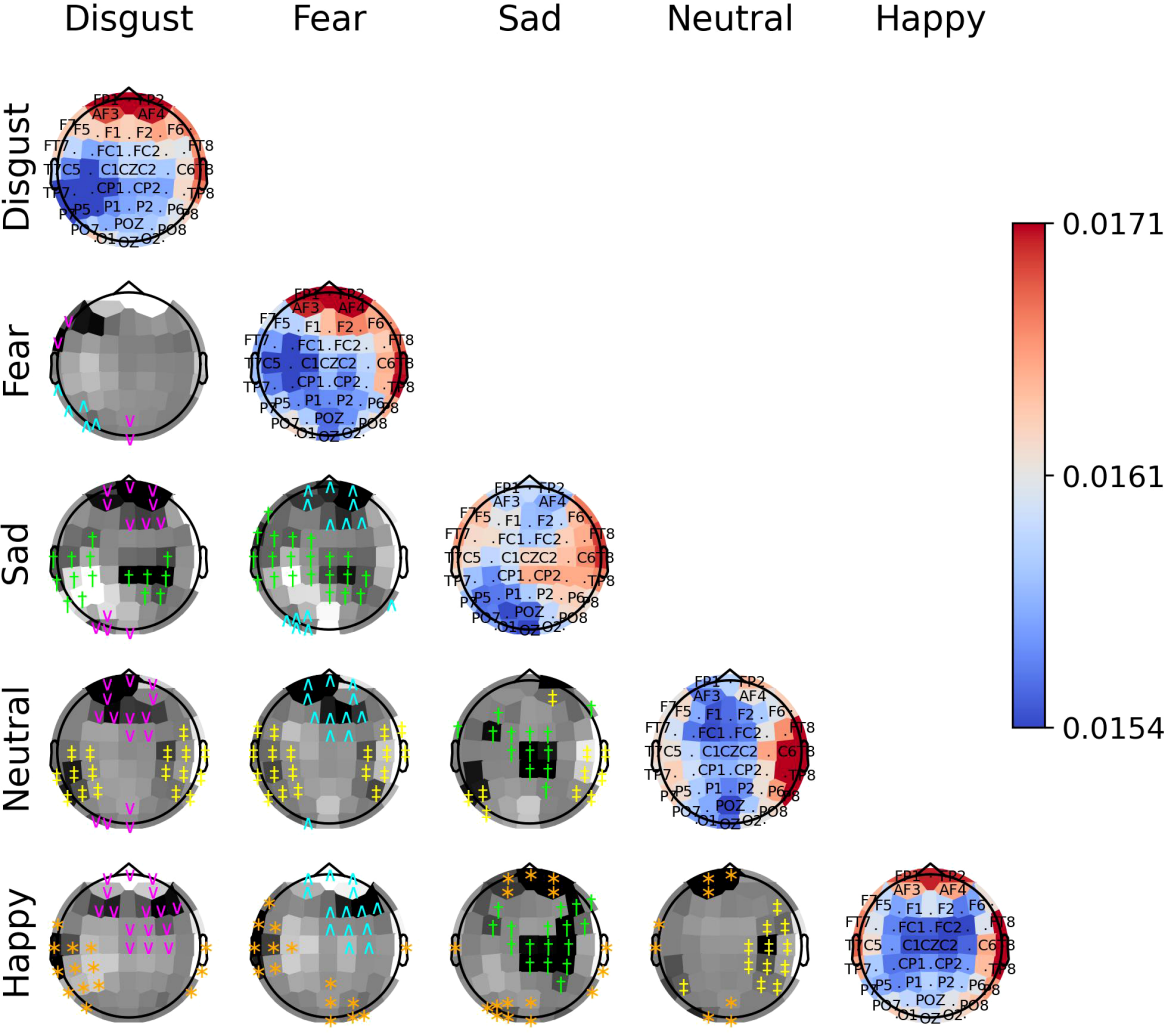
The diagonal panels shows aggregated attention weights obtained by the EEG channels for SEED-V. The off-diagonal panels are the difference between aggregated attention weights obtained by the EEG channels. A darker color indicates a greater difference between the aggregated weights obtained for the EEG channel for the vertical and horizontal emotion pairs. Each symbol indicates that the weight difference between the emotion pair was significant (2-sided Wilcoxon rank-sum hypothesis tests adjusted via Benjamini-Hochberg correction with a false-positive rate set at 0.05) in favor of the class ‘disgust’ (magenta ^∨^), ‘fear’ (cyan ^∧^), ‘sad’ (green †), ‘neutral’ (yellow ‡), or ‘happy’ (orange ∗).

The diagonal panels of **Figures 8**–**10** display the aggregated attention weights for each emotion. For all emotions, the aggregated attention weights were more pronounced along the circumference of the head, particularly over the prefrontal, frontal, fronto-temporal, temporal, temporal-parietal, parietal, and parietal-occipital EEG regions.

The off-diagonal panels of **Figures 8**–**10** show the differences in attention weights between each pair of emotions. The attention weights varied significantly among the emotion pairs, mainly in the frontal, temporal, parietal, and occipital regions (2-sided Wilcoxon rank-sum hypothesis tests, adjusted via Benjamini-Hochberg correction with a false discovery rate set at 0.05). Although there was not complete agreement between the differences found for the emotion pairs in the SEED, SEED-IV and SEED-V datasets, the observed differences suggest common trends for some emotions. For example, the sad emotion exhibited higher attention weights around the middle parietal and occipital regions of the cortex compared to other emotions. Similarly, the neutral class showed dominance over other emotions in the right frontal, temporal, and parietal regions.

In SEED-IV and SEED-V, the fear, disgust, and happy classes tended to have higher attention weights in the frontal and temporal areas than the neutral and sad classes. However, when comparing the attention weights for fear and happy between SEED-IV and SEED-V, an opposite pattern emerged. In SEED-IV, the fear class had higher weights than happy in the left temporal-parietal area, whereas happy had higher values in the right frontal-temporal area than fear. In contrast, in SEED-V, the fear class exhibited higher weights in the right frontal-temporal area than happy, while happy showed higher weights in the left temporal-parietal area.

## 4 Discussion

### 4.1 Main findings

Our findings indicate that the EEG channels that provide the most relevant features for emotion prediction across individuals are those located along the head circumference. Specifically, features extracted from channels *Fp*_1_, *Fp*_2_, *F*_7_, *F*_8_, *FT*_7_, *FT*_8_, *T*_7_, *T*_8_, *TP*_7_, *TP*_8_, *P*_7_, *P*_8_, *PO*_7_, *PO*_8_, *O*_1_, and *O*_2_ contribute the most to emotion prediction throughout stimuli exposure. The attention weights from the channels show significant variations across different emotional states, demonstrating their ability to distinguish between different emotional responses. Thus, capturing electrical activity from this region is essential for enhancing the prediction of emotions elicited by audiovisual stimuli in subject-independent methodologies.

Regarding the emotion recognition performance, the attention network-based model achieved an average accuracy of 79.3%, 69.5% and 60.7 for SEED, SEED-IV and SEED-V, respectively. These accuracy rates are comparable to those of previous studies using SEED-IV and SEED-IV (see **Table 7**), thus showing that the proposed deep learning architecture was able to extract common patterns shared between different subjects. The low performance for some subjects is also consistent with Li et al. (19), who reported that subjects 5 and 10 in SEED-V resulted in the lowest accuracy performance compared to the remaining subjects.

The ablation study (refer to **Table 8**) highlighted the significance of spatial and temporal processing components in emotion recognition. This relevance arises from the use of video clips to elicit emotions. Given that the EEG signal responds dynamically to the varying scenes within the video, it is crucial to incorporate components that effectively capture this information from the EEG channels, along with its progression over time.

The importance of EEG channels located in the frontal, parietal, temporal, and occipital regions, as indicated by the attention mechanism weights, aligns with existing psychological literature on brain function (44). Specifically, since the stimuli were audiovisual, features extracted from EEG channels in sensory brain areas (temporal and parietal for audio and occipital for visual) played a relevant role in emotion prediction (44–46). When viewing videos, the temporal, parietal, and occipital regions are activated to process audiovisual content, including facial expressions, body language, speech, and sounds that convey emotions (47). Moreover, the shift of activation weights from temporal and occipital regions to frontal regions (see **Figures 2**–**4**) suggests that once relevant audiovisual information is captured by sensory areas, it is subsequently processed in the frontal and prefrontal regions (48).

By comparing the identified EEG channels with those from commercial EEG systems designed for emotion monitoring, such as the EMOTIV EPOC X 14-channel wireless headset (49), we observe a notable overlap among the channels. Specifically, the 14 EEG channels included in the EPOC X system are primarily located along the head circumference (*AF*_3_, *AF*_4_, *F*_3_, *F*_4_, *F*_7_, *F*_8_, *FC*_5_, *FC*_6_, *P*_7_, *P*_8_, *T*_7_, *T*_8_, *O*_1_, and *O*_2_). Thus, our study offers evidence supporting the reliability of these lower-density EEG channel systems for recognizing emotions evoked by audiovisual stimuli.

Identifying relevant EEG channels enables the development of EEG-based emotion recognition systems with fewer channels. Such systems can be more usable, such as a headset with fewer EEG channels, which is more convenient and comfortable to wear. This could be beneficial for individuals with neurological diseases or older adults, which require frequent neural monitoring for early diagnosis, intervention, and treatment.

### 4.2 Comparison with previous studies

Similar to Apicella et al. (23), our study also indicates that prefrontal and frontal EEG channels are relevant for predicting emotions. Additionally, consistent with previous research that analyzed entropy distribution differences by emotion, our results highlight the lateral temporal lobe and prefrontal lobe as critical regions for extracting features for emotion prediction. However, unlike these earlier studies, we are, to the best of our knowledge, the first to analyze learned patterns of a deep learning model to provide evidence on the specific EEG channels that contribute most significantly to emotion prediction. Furthermore, we conduct our analysis using a subject-independent approach across two different datasets, supporting the reproducibility and generalizability of our findings. These results underscore the importance of incorporating features from EEG channels located along the head circumference to enhance emotion prediction in subject-independent scenarios for emotions evoked by audiovisual stimuli.

Regardless of the emotion type, the attention weights reveal that features extracted from both brain hemispheres are relevant for predicting emotions (see **Figures 8**–**10**). This is in contrast to previous studies (50, 51) that suggested brain lateralization in emotion processing, where negative emotions are primarily processed in the right hemisphere and positive emotions in the left. In our findings, we did not observe distinct roles for each hemisphere in emotion prediction. Instead, the predictive model relied on features extracted from EEG channels located in the frontal, parietal, temporal, and occipital regions along the head circumference from both hemispheres, underscoring the importance of both the left and right hemispheres in predicting any emotion type.

Although the predictive model did not rely too much on features extracted from the central EEG channels, the central and central-parietal channels (*C_Z_*, *CP_Z_*) were found to be relevant for the sad emotion in both datasets. Given that the sad class is the only emotion categorized as low arousal according to the valence-arousal model of emotions (52), this finding suggests that the temporal-spatial features extracted from central EEG channels may be particularly important for predicting emotions with low arousal.

### 4.3 Limitations and future work

We note that our experiments were conducted using datasets (SEED, SEED-IV and SEED-V) that encompass subjects from a similar population (20-to 24-year-old undergraduate students at Shanghai Jiao Tong University). Given that EEG data vary among individuals due to factors such as culture, language, and genetics (4, 53), our findings may not be universally applicable to individuals from different backgrounds. For instance, studies have shown that cultural differences between Western and Asian populations can affect the performance of emotion recognition methods (54). However, despite the fact that the SEED, SEED-IV and SEED-V datasets were collected at the same location, the 46 subjects in each dataset were mutually exclusive, ensuring fair validation of our study results. Moreover, the 95% confidence interval for the average accuracy suggests potential generalizability to other datasets. Future research should validate these results across diverse datasets encompassing broader emotional states and subjects.

We also recognize that the current study focused on emotion datasets featuring discrete emotions (e.g., happy, sad), and our model has not yet been evaluated on datasets utilizing the arousal-valence model. Therefore, future research should consider extending our work to classify emotions based on their arousal and valence levels, which may offer valuable insights into the neuronal patterns associated with these emotional dimensions.

## 5 Conclusion

This study presents a deep learning model with attention mechanism layers to identify the EEG channels most relevant to emotion prediction. The attention weights revealed that the model predominantly relied on features extracted from EEG channels located along the head circumference, which cover sensorimotor areas (temporal, parietal, and occipital) as well as the frontal regions. Additionally, the attention weights of these channels varied significantly across emotions, demonstrating their potential for distinguishing emotional states. Thus, EEG channels along the head circumference are crucial for capturing the relevant electrical activity that aids in predicting emotions evoked by audiovisual stimuli in subject-independent approaches.

## Data Availability

The original contributions presented in the study are included in the article/**Supplementary Material**. Further inquiries can be directed to the corresponding author.

## Supplementary material

SUPPLEMENTARY FIGURE 1 Average spatial attention weights ( $\bar{\phi }$ *_k,s_*) for the 62 EEG channels across the 15 subjects of the SEED for each emotion and each of the 66 four-second windows.

SUPPLEMENTARY FIGURE 2 Average spatial attention weights ( $\bar{\phi }$ *_k,s_*) for the 62 EEG channels across the 15 subjects of the SEED-IV for each emotion and each of the 63 four-second windows.

SUPPLEMENTARY FIGURE 3 Average spatial attention weights ( $\bar{\phi }$ *_k,s_*) for the 62 EEG channels across the 16 subjects of the SEED-V for each emotion and each of the 74 four-second windows.
