# Supplementary figures and images for: Identifying relevant EEG channels for subject-independent emotion recognition using attention network layers
Source: Front Psychiatry. 2025 Feb 10;16:1494369. doi: 10.3389/fpsyt.2025.1494369 (PMC11847823; doi:10.3389/fpsyt.2025.1494369)

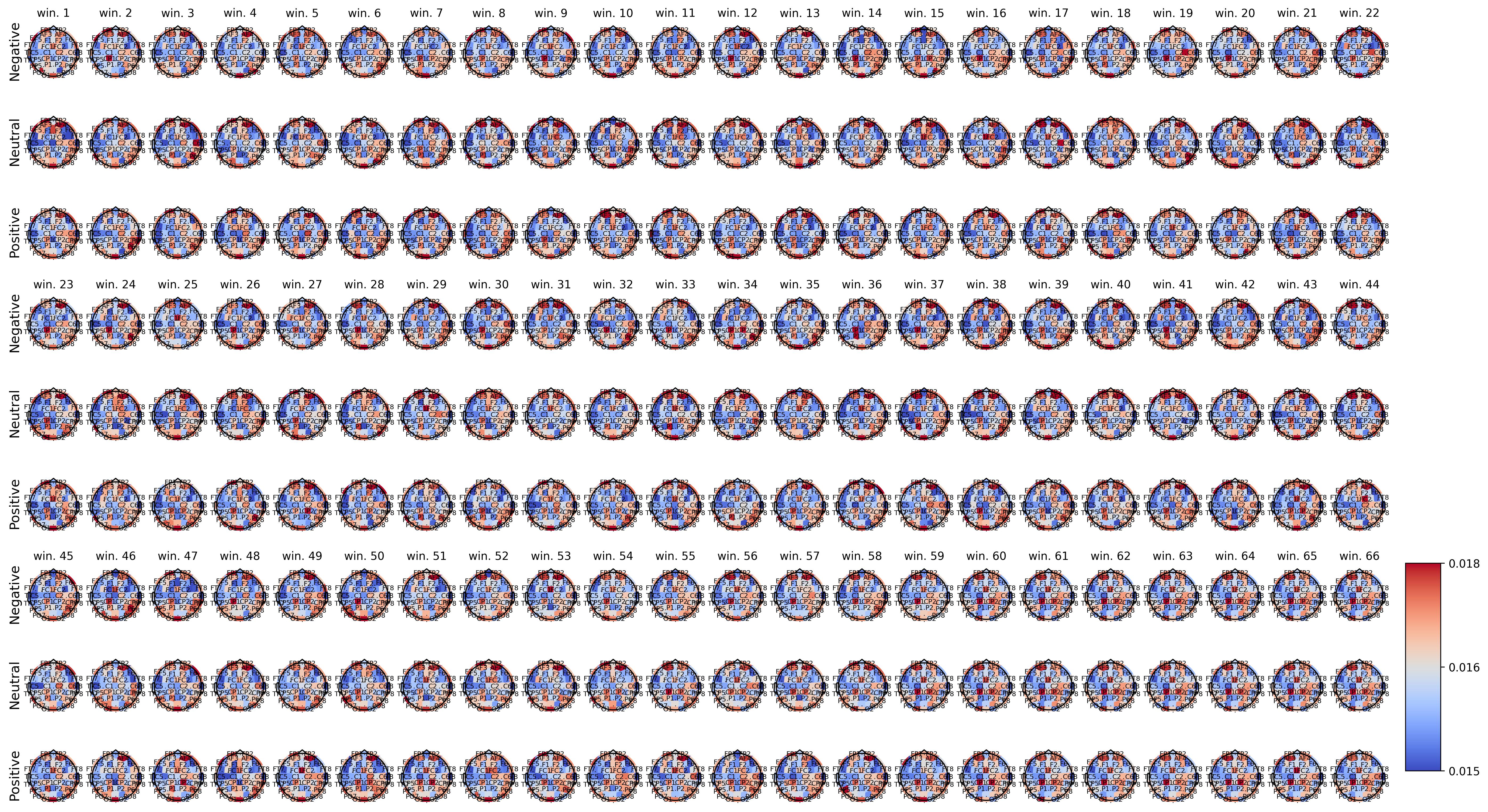

Supplement: SUPPLEMENTARY FIGURE 1 — Average spatial attention weights ( ϕ¯ k,s ) for the 62 EEG channels across the 15 subjects of the SEED for each emotion and each of the 66 four-second windows. [file Image1.jpeg]

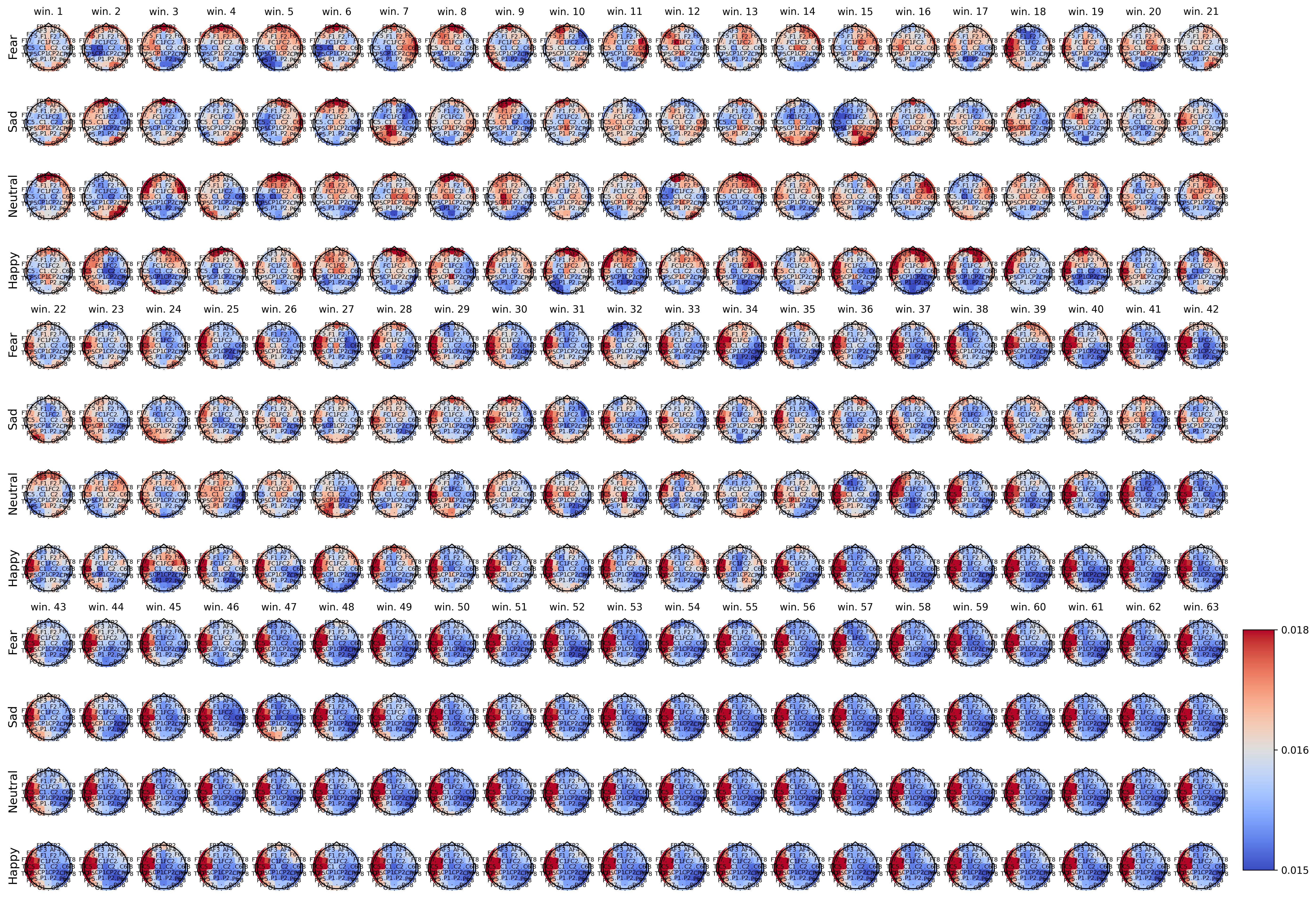

Supplement: SUPPLEMENTARY FIGURE 2 — Average spatial attention weights ( ϕ¯ k,s ) for the 62 EEG channels across the 15 subjects of the SEED-IV for each emotion and each of the 63 four-second windows. [file Image2.jpeg]

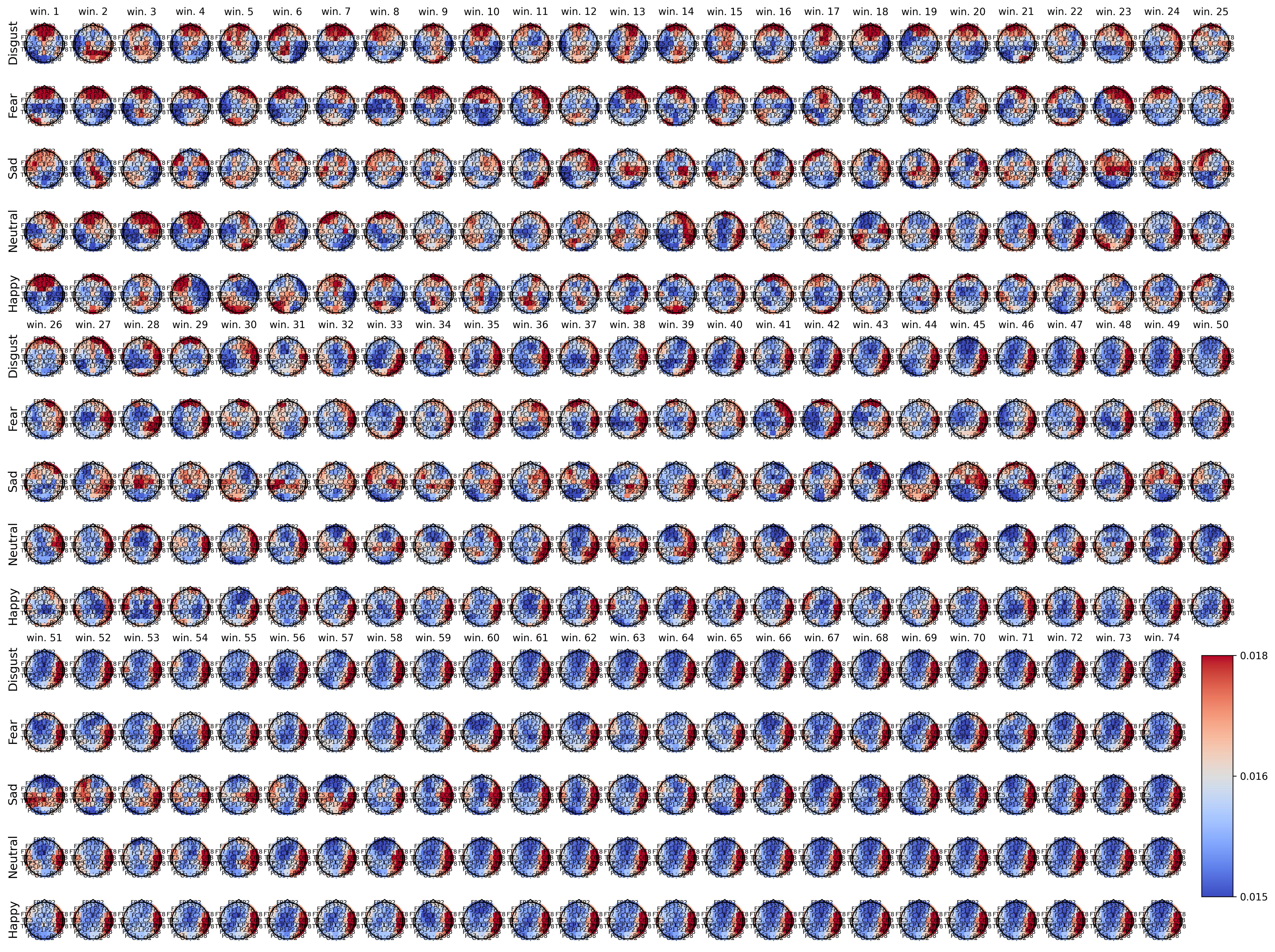

Supplement: SUPPLEMENTARY FIGURE 3 — Average spatial attention weights ( ϕ¯ k,s ) for the 62 EEG channels across the 16 subjects of the SEED-V for each emotion and each of the 74 four-second windows. [file Image3.jpeg]
